# Granger Causality on forward and Reversed Time Series

**DOI:** 10.3390/e23040409

**Published:** 2021-03-30

**Authors:** Martina Chvosteková, Jozef Jakubík, Anna Krakovská

**Affiliations:** Institute of Measurement Science, Slovak Academy of Sciences, 84104 Bratislava, Slovakia; jozef.jakubik@savba.sk (J.J.); krakovska@savba.sk (A.K.)

**Keywords:** time reversal, Granger causality, predictive error, endogeneity, 05.45.Tp

## Abstract

In this study, the information flow time arrow is investigated for stochastic data defined by vector autoregressive models. The time series are analyzed forward and backward by different Granger causality detection methods. Besides the normal distribution, which is usually required for the validity of Granger causality analysis, several other distributions of predictive errors are considered. A clear effect of a change in the order of cause and effect on the time-reversed series of unidirectionally connected variables was detected with standard Granger causality test (GC), when the product of the connection strength and the ratio of the predictive errors of the driver and the recipient was below a certain level, otherwise bidirectional causal connection was detected. On the other hand, opposite causal link was detected unconditionally by the methods based on the time reversal testing, but they were not able to detect correct bidirectional connection. The usefulness of the backward analysis is manifested in cases where falsely detected unidirectional connections can be rejected by applying the result obtained after the time reversal, and in cases of uncorrelated causally independent variables, where the absence of a causal link detected by GC on the original series should be confirmed on the time-reversed series.

## 1. Introduction

Investigating causal relations between simultaneous recordings of variables is a common task in scientific fields as diverse as neuroscience [1], climatology [2], and economy [3]. In 1969, Clive Granger proposed a testable definition of causality between two processes *X* and *Y* based on predictability and precedence [4]. As all available information, he considered knowledge of two stationary time series, *x* and *y*, corresponding to variables *X* and *Y*, respectively. If the predictive error variance of *y* only from past *y* values is greater than the predictive error variance of *y* from both past *x* and past *y* values, then the variable *X* is said to cause variable *Y*, denoted X→Y. Granger suggested to use linear autoregressive (AR) predictor, which is simple to interpret and mathematically easy to handle. The standard Granger causality test (GC) refers to an *F*-test for significance of regression coefficients.

A slightly different approach to test Granger causality that we will also use here is to test for predictive errors (PEGC) instead of testing for regression coefficients. It means that the null hypothesis of no predictability improvement is statistically tested against the alternative hypothesis that the inclusion of the knowledge of *x* significantly improves the prediction of *y* (causal connection from X to Y). Analogously, we test the opposite direction Y→X. We adopted the approach from the predictability improvement method designed as a generalization of the GC test for reconstructed state spaces [5].

To avoid the problem of spurious causal detections, especially in the analysis of electroencephalographic signals, Haufe et al. [6] have suggested using the time-reversed series as surrogate data and called this procedure time-reversed Granger causality (TRGC) [7]. They have proposed to contrast a value of the net Granger score [8,9] obtained from the original data against a value of the net Granger score obtained from the time-reversed data. The time-reversed data, as a special case of possible permutation of the data, represents the surrogate data for which weak asymmetries are preserved and strong asymmetries are exactly inverted [6]. Using simulations, it has been shown that TRGC robustly rejects causal interpretations on mixtures of independent processes [6], and can indicate the correct direction of causal interaction in the case of unidirectionally linearly connected autoregressive processes [7]. However, TRGC by definition is not able to detect a so-called feedback, i.e., bidirectional causal connection between variables. Only the predominant direction of information flow between two variables can be detected dealing with the net-GC and time inversion testing. In this study, the performance of a proposed modification of TRGC (mTRGC), which also allows the detection of a feedback, is investigated.

The concept of time inversion testing is based on the intuitive idea, that if the first principle of Granger causality that the cause precedes the effect holds, then reversed role between a driver and its recipient can be expected for the time-reversed series, but can we really expect that? In [10], Paluš et al., investigating the role of the time arrow in coupled irreversible processes, have found some surprising results. For example, for the case of bivariate order-one AR model with unidirectional connection, the standard GC failed to detect unidirectional but reversed causality when analyzing time reversed series. Instead, the method resulted in detection of bidirectional connection.

In this paper, Granger’s analysis of causality between two variables in the context of time reversals is numerically studied. We are mainly interested in the effect of time reversal on the change in the order of cause and effect. Three different Granger causality detection methods are used. They are applied to linear autoregressive processes for which the Granger’s causality is originally formulated. According to the literature, the validity of the *F*-test for Granger causality is only guaranteed for the normally distributed predictive errors of present values *x* and *y*, see e.g., [11]. In this study, we decided to consider different distributions of predictive errors and analyze the effect of the errors term’s distribution on causality testing both for the original time-ordered and the time-reversed series.

As we have already indicated, in addition to the effect of predictive error distribution, we are also interested in whether the type of used causal method plays a role. To find out, we numerically tested several ways to estimate Granger causality.

Granger causality and three approaches for testing Granger causality are introduced in Section 2. Data and the experimental setup for our simulation study are described in Section 3. Results are summarized in Section 4 and the discussion is given in Section 5.

## 2. Methods

In the context of bivariate Granger causality, we will consider two variables *X* and *Y*, represented by simultaneously observed stationary zero mean time series x:={x(1) , x(2),…,x(T)} and y:={y(1) , y(2),…,y(T)}, respectively. The causal analysis from a driving variable *X* to a response variable *Y* involves two linear models [4]. The first one is a bivariate autoregressive model

(1) $$x\left(t\right)=\sum_{i=1}^{p}a_{xx,i}x\left(t-i\right)+\sum_{j=1}^{p}a_{xy,j}y\left(t-j\right)+\epsilon_{xy}\left(t\right)$$

(2) $$y\left(t\right)=\sum_{i=1}^{p}a_{yx,i}x\left(t-i\right)+\sum_{j=1}^{p}a_{yy,j}y\left(t-j\right)+\epsilon_{yx}\left(t\right),$$

where $a_{xx,j}$, $a_{xy,j}$, $a_{yx,j}$, and $a_{yy,j}$ are coefficients of the model; and ${\left(\epsilon_{xy},\epsilon_{yx}\right)}^{{}^{\prime }}$ is a 2-dimensional unobservable zero mean white noise process with time invariant covariance matrix ∑. The dependence of *y* on the past *x* in the linear autoregressive model (2), given its own past, is encapsulated in the coefficients $a_{yx,i}$. The consideration that there is no dependence of *y* on the past of *x* leads to the second model

(3) $$y\left(t\right)=\sum_{j=1}^{p}a_{y,j}y\left(t-j\right)+\epsilon_{yy}\left(t\right),$$

where $a_{y,j}$ are AR coefficients; and predictive error (or residuals) $\epsilon_{yy}$ is white noise process with a variance $\sigma_{y}^{2}$. If the past of *x* is found to be helpful for predicting *y*, then *X* is said to Granger-cause *Y*; otherwise *X* is said to fail to Granger-cause *Y*.

### 2.1. The Standard Granger Causality Test (GC)

Variable *X* fails to Granger-cause *Y* if all $a_{yx,i}$ coefficients are zero. A parametric statistical significance test on the regression coefficients, i.e., $H_{0}:a_{yx,1}=\ldots =a_{yx,p}=0$, is usually provided with the Fisher test statistic

(4) $$F^{X\to Y}=\frac{(SSR_{y}^{H_{0}}-SSR_{yx})/p}{SSR_{yx}/\left(T-3p\right)}{∼}_{as.}F_{p,T-3p},$$

where $SSR_{y}^{H_{0}}$ is the sum of squared residuals $\epsilon_{yy}\left(t\right)$ from the regression model (3) restricted by the null hypothesis $H_{0}$, and $SSR_{yx}$ is the sum of squared residuals $\epsilon_{yx}\left(t\right)$ from the full (or unrestricted) model (2). Under the null hypothesis the test statistic (4) has an asymptotic *F*-distribution with *p* and T−3p degrees of freedom. If $F^{X\to Y}$ is greater than a quantile of $F_{p,T-3p}$-distribution at a chosen significance level, then the null hypothesis is rejected and it is concluded that *X* Granger-causes *Y*. To search for the causal influence in the opposite direction, i.e., Y→X, the values $SSR_{y}^{H_{0}}$ and $SSR_{yx}$ in (4) are replaced by $SSR_{x}^{H_{0}}$ and $SSR_{xy}$, respectively. The value $SSR_{x}^{H_{0}}$ is the sum of squared residuals $\epsilon_{xx}\left(t\right)$ from the regression model

(5) $$x\left(t\right)=\sum_{j=1}^{p}a_{x,j}x\left(t-j\right)+\epsilon_{xx}\left(t\right)$$

restricted by the null hypothesis $H_{0}:a_{xy,1}=\ldots =a_{xy,p}=0$, and $SSR_{xy}$ is the sum of squared residuals $\epsilon_{xy}\left(t\right)$ from the full model (1).

The regression coefficients in (1)–(5) may be estimated separately by ordinary least squares (OLS). The whiteness of predictive errors is a crucial assumption for a valid causal analysis. Autocorrelation of the predictive errors implies that also regressors and the predictive errors are correlated. As a result, the regression coefficient estimates fail to converge to the true value of the regression coefficients as sample size increases. This bias is referred to as the endogeneity bias and may affect the Granger causality inference [12]. The problem with identification of a vector autoregressive model (VAR) also arises in the presence of instantaneous interactions between variables. Such interactions can occur in practice if the sampling rate of the records falls below the time scale of causal interactions. This can lead to a falsely detected feedback. There is no instantaneous causality if and only if the vector predictive errors ${\left(\epsilon_{xy},\epsilon_{yx}\right)}^{{}^{\prime }}$ have uncorrelated components. Such predictive errors are often called innovations [13]. Granger causality inference is valid only if autoregressive models can adequately capture the correlation structure in the data.

The order *p* of VAR can be determined using a model selection criterion. For example, the Akaike information criterion [14] and the Schwartz–Bayesian information criterion [15] are commonly used to estimate the order. An *F*-test for testing the submodel is meaningful if both the full and the restricted models are well-defined linear models. In fact, while the full model is of finite order, the reduced one is generally of infinite order. To eliminate potentially problematic consequences for Granger causality analysis, it can be recommended to estimate appropriate model order for the reduced model, rather than for the full model [16].

### 2.2. Predictive Error Test for Granger Causality (PEGC)

If all coefficients $a_{yx,j}$, j=1,…,p are zero, then it is stated that *X* does not Granger cause *Y*. This seems to fit definition of no Granger causality, when the variance of predictive error of *y* using only past of *y* cannot be reduced by also using the past of *x* [4]. The predictability improvement, a nonparametric generalization of Granger causality in reconstructed state spaces, evaluates a causal connection between variables by testing the equality of predictive errors [5,17]. Here, we adopt the approach such that, instead of testing regression coefficients, the causal link X→Y is analyzed by comparing the predictive errors $\epsilon_{yy}$, $\epsilon_{yx}$ and the causal link Y→X is analyzed by comparing the predictive errors $\epsilon_{xx}$, $\epsilon_{xy}$. If the null hypothesis of the absence of a causal link X→Y, i.e., $H_{0}:\epsilon_{yy}=\epsilon_{yx}$, is rejected against the alternative that the prediction of *y* is significantly improved by including the information of past *x* in a linear autoregressive prediction, i.e., $H_{A}:\epsilon_{yy}>\epsilon_{yx}$, on a significance level, then it is concluded that *X* causes *Y*. Analogous testing procedure is applied to analyze the causal connection Y→X.

### 2.3. Modification of Time-reversed Granger Causality Test (mTRGC)

The additional information contained in variable *X* about the future value of variable *Y*, and in *Y* about the future of *X*, is quantified by the Granger causality score [7,18] defined as

(6) $$G_{X\to Y}=log\left(SSR_{y}^{H_{0}}/SSR_{yx}\right)\;\;and\;\;G_{Y\to X}=log\left(SSR_{x}^{H_{0}}/SSR_{xy}\right),$$

respectively. Larger values of $G_{X\to Y}$ indicate that the past of *X* helps to improve the prediction of *Y*. On the other hand, the values of $G_{X\to Y}$ close to zero indicate that the past of *X* does not improve prediction of *y*, meaning that *X* does not Granger cause *Y*.

Let ${\left(\tilde{x}\left(t\right),\tilde{y}\left(t\right)\right)}^{{}^{\prime }}$ denotes the time-reversed bivariate autoregressive process (i.e., $(\tilde{x}\left(t\right),$ $\tilde{y}{\left(t\right))}^{{}^{\prime }}={\left(x\left(T-t+1\right),y\left(T-t+1\right)\right)}^{{}^{\prime }}$). The difference based TRGC [6,7] analyzes a causal interaction between *X* and *Y* using the difference of the net Granger scores obtained from the original data, given as $G_{X\to Y}-G_{Y\to X}$, and the net Granger scores obtained from the time-reversed data, given as $G_{\tilde{X}\to \tilde{Y}}-G_{\tilde{Y}\to \tilde{X}}$, where $G_{\tilde{X}\to \tilde{Y}}$, $G_{\tilde{Y}\to \tilde{X}}$ are the Granger scores computed on $\tilde{x},\tilde{y}$. The presence of causal connection X→Y is detected by TRGC if $G_{X\to Y}-G_{Y\to X}$ is significantly greater than $G_{\tilde{X}\to \tilde{Y}}-G_{\tilde{Y}\to \tilde{X}}$, the opposite causal connection Y→X is detected if $G_{X\to Y}-G_{Y\to X}$ is significantly less than $G_{\tilde{X}\to \tilde{Y}}-G_{\tilde{Y}\to \tilde{X}}$, and the absence of a causal connection between variables X,Y is concluded if there is no statistically significant difference between the net scores. We see that TRGC is by definition unable to detect the bidirectional causal connection between variables.

Winkler et al. [7] also showed that if *X* Granger causes *Y* and *Y* does not Granger cause *X*, then $D_{X\to Y}\geq 0$, $D_{Y\to X}\leq 0$ for infinite samples, where the variables $D_{X\to Y}$, $D_{Y\to X}$ are defined as

(7) $$D_{X\to Y}=G_{X\to Y}-G_{\tilde{X}\to \tilde{Y}}\;\;\mathrm{and}\;\;D_{Y\to X}=G_{Y\to X}-G_{\tilde{Y}\to \tilde{X}}.$$

Instead of the net Granger scores, we propose to examine the difference variable $D_{X\to Y}$ and $D_{Y\to X}$ for investigating causal relation between X,Y. Namely, the causal connection X→Y is detected if $D_{X\to Y}$ is greater than zero, otherwise it is concluded that *X* does not Granger cause *Y*. Analogously, the causal connection Y→X is detected if $D_{Y\to X}$ is greater than zero, otherwise it is concluded that *Y* does not Granger cause *X*. We see that with this modification, we should also be able to detect bidirectional connection. Similarly to TRGC, the bootstrapping approach can be applied to perform statistical inference [19].

We propose two versions of TRGC modification. The first one includes a statistical significance testing and is denoted as mTRGC. The second version is based on non-statistical evaluation of $D_{X\to Y}$, $D_{Y\to X}$ and is denoted as mTRGC*.

In addition, we test the combination of GC and mTRGC*, denoted GC+mTRGC*. A causal link is detected by GC+mTRGC*, if the causal link is found to be significant by GC and the detection is confirmed by mTRGC* subsequently.

The introduced methods GC, PEGC, mTRGC, mTRGC*, and GC+mTRGC* will be applied to detection of causal interaction between two variables in numerical experiments without an influence of a common hidden variable, and measurement noise. The performance of all five methods is numerically examined on processes generated by a bivariate order-one AR model under considering seven different distributions of the predictive errors. Besides the normal distribution typically used for defining VAR, serially independent predictive errors are generated by a uniform distribution, triangular distribution, and a mixture of normal distributions. In addition, the predictive errors generated by the moving-average model, and quadratic moving-average model is used to analyze the impact of model assumption violations to the performance of the Granger causality detection methods. Moreover, the effect of instantaneous interactions is analyzed through generating correlated predictive errors. Causal relationship will be analyzed by all introduced methods on both originally generated time series and the time-reversed series.

## 3. Data and Experimental Setup

Through the numerical experiments in this study, the performance of the bivariate Granger causality detection methods was investigated. A causal interaction was analyzed on a pair of known causal structure processes with original temporal order and with reversed temporal order. Three types of causal relationships between the two variables X and Y were considered: causal independence (X⊥Y), unidirectional causal link (X→Y), and bidirectional causal link (X↔Y). The corresponding series were generated by a simple linear autoregressive model with the predictive error of various distributions. The model systems were as follows:

**Causal independence (X⊥Y)**
  (8) $$\begin{array}{cc} x(t)= & 0.5x\left(t-1\right)+\epsilon_{x}\left(t\right)\\ y(t)= & ay\left(t-1\right)+\epsilon_{y}\left(t\right), \end{array}$$
  where 19 values of *a* were considered, a∈{0.05,0.10,…,0.95}.
**Unidirectional causal connection (X→Y)**
  (9) $$\begin{array}{cc} x(t)= & 0.5x\left(t-1\right)+\epsilon_{x}\left(t\right)\\ y(t)= & 0.5y\left(t-1\right)+c_{1}x\left(t-1\right)+\epsilon_{y}\left(t\right), \end{array}$$
  where 49 values of $c_{1}$ were considered, $c_{1}\in \left\{0.02,0.04,\ldots ,0.98\right\}$.
**Bidirectional causal connection (X↔Y)**
  (10) $$\begin{array}{cc} x(t)= & 0.5x\left(t-1\right)+0.5y\left(t-1\right)+\epsilon_{x}\left(t\right)\\ y(t)= & 0.5y\left(t-1\right)+c_{2}x\left(t-1\right)+\epsilon_{y}\left(t\right), \end{array}$$
  where 19 values of $c_{2}$ were considered, $c_{2}\in \left\{0.025,0.05,\ldots ,0.475\right\}$. The connectivity structure of the model systems was controlled by parameters $c_{1},c_{2}$.

The predictive errors $\epsilon_{x}$, $\epsilon_{y}$ were generated under seven different conditions:

Condition A (normal distribution): The predictive errors $\epsilon_{x}$, $\epsilon_{y}$ were independent normally distributed random variables with zero mean and with the variance $\sigma_{x}^{2}=0.5$ and $\sigma_{y}^{2}=\sigma_{x}^{2}*\left\{0.25,0.5,0.75,1,1.25,1.5,1.75\right\}$ (i.e., $\sigma_{y}^{2}$ was a multiple of $\sigma_{x}^{2}$), respectively.
Condition B (uniform distribution): The predictive errors $\epsilon_{x}$, $\epsilon_{y}$ were independent uniformly distributed random variables in intervals $\left[a_{x},b_{x}\right]$, $\left[a_{y},b_{y}\right]$, respectively. The distribution parameters for $\epsilon_{x}$ were: $a_{x}=-\sqrt{3}/2$, and $b_{x}=-a_{x}$. The distribution parameters for $\epsilon_{y}$ were: $a_{y}=a_{x}*\left\{0.25,0.5,0.75,1,1.25,1.5,1.75\right\}$, and $b_{y}=-a_{y}$.
Condition C (triangular distribution): The predictive errors $\epsilon_{x}$, $\epsilon_{y}$ were independent triangular-distributed random variables. The triangular distribution parameters for $\epsilon_{x}$ were: lower limit $a_{x}=-2$, upper limit $b_{x}=-a_{x}/2$ and mode $c_{x}=b_{x}$. The triangular distribution parameters for $\epsilon_{y}$ were: lower limit $a_{y}=a_{x}*\left\{0.25,0.5,0.75,1,1.25,1.5,1.75\right\}$, upper limit $b_{y}=-a_{y}/2$ and mode $c_{y}=b_{y}$.
Condition D (a mixture of normal distributions): Both predictive errors $\epsilon_{x}$, $\epsilon_{y}$ were generated from a mixture of two normal distributions. The error term $\epsilon_{x}$ was generated from a distribution where the probability of drawing from the normal distribution $N(1,5\left(\sigma_{x}^{2}-1/4\right)/9)$ was 1/5 and from the normal distribution $N(-1/4,10\left(\sigma_{x}^{2}-1/4\right)/9)$ was 4/5, where $\sigma_{x}^{2}=0.5$. The error term $\epsilon_{y}$ was generated from a distribution where the probability of drawing from the normal distribution $N(1,5\left(\sigma_{y}^{2}-1/4\right)/9)$ was 1/5 and from the normal distribution $N(-1/4,10\left(\sigma_{y}^{2}-1/4\right)/9)$ was 4/5, where $\sigma_{y}^{2}=\sigma_{x}^{2}*\left\{0.75,1,1.25,1.5,1.75\right\}$.
Condition E (moving average): The predictive errors $\epsilon_{x}$, $\epsilon_{y}$ were defined as $\epsilon_{x}\left(t\right)=0.5\xi_{x}\left(t-1\right)+\xi_{x}\left(t\right)$, $\epsilon_{y}\left(t\right)=0.5\xi_{y}\left(t-1\right)+\xi_{y}\left(t\right)$, respectively. The variables $\xi_{x}$, $\xi_{y}$ were independent normally distributed with zero mean and with the variance $\sigma_{x}^{2}=0.4$ and $\sigma_{y}^{2}=\sigma_{x}^{2}*\left\{0.25,0.5,0.75,1,1.25,1.5,1.75\right\}$, respectively.
Condition F (quadratic moving average): The predictive errors $\epsilon_{x}$, $\epsilon_{y}$ were defined as $\epsilon_{x}\left(t\right)=0.5\xi_{x}^{2}\left(t-1\right)-0.5\xi_{x}^{2}\left(t\right)$, $\epsilon_{y}\left(t\right)=0.5\xi_{y}^{2}\left(t-1\right)-0.5\xi_{y}^{2}\left(t\right)$, respectively. The variables $\xi_{x}$, $\xi_{y}$ were independent normally distributed with zero mean and with the variance $\sigma_{x}^{2}=\sqrt{0.5}$ and $\sigma_{y}^{2}=\sigma_{x}^{2}*\left\{0.25,0.5,0.75,1,1.25,1.5,1.75\right\}$, respectively.
Condition G (correlation): The predictive errors $\epsilon_{x}$, $\epsilon_{y}$ were correlated, with $cov(\epsilon_{x},\epsilon_{y})$ = 0.1. Like in the condition A, the error terms were normally distributed variables with zero mean and with the variance $\sigma_{x}^{2}=0.5$ and $\sigma_{y}^{2}=\sigma_{x}^{2}*\left\{0.25,0.5,0.75,1,1.25,1.5,1.75\right\}$, respectively.

Note that various parameters in conditions B–G were chosen to obtain the same means and variances of variables $\epsilon_{x}$, $\epsilon_{y}$ as set in condition A. Only in the condition D, the first two values of $\sigma_{y}^{2}$ had to be omitted due to the requirement $(\sigma_{y}^{2}-1/4)>0$ in the variance of $\epsilon_{y}$. The random variables $\epsilon_{x}$, $\epsilon_{y}$ were serially uncorrelated for conditions A-D, and serially correlated for conditions E–F. In the condition G, the residuals were correlated with each other.

The investigation of causal interaction between variables was performed with generated time series of length T={300,3000} for all combinations of model systems and conditions, after the initial $10^{4}$ iterations were discarded for each dataset. The experiments were repeated 500 times. Two separate GC tests, two separate PEGC tests, two separate mTRGC tests, two separate mTRGC* tests, and two separate GC+mTRGC* tests were performed (one for X→Y, one for Y→X) on the originally generated series and on the time-reversed series. The statistical tests detected a causal link at the significance level α/2 with α=0.05.

Instead of a bootstraping method, the (1−α/2)-confidence intervals on the difference variables $D_{X\to Y}$ and $D_{Y\to X}$ for evaluating mTRGC were constructed by using the $D_{X\to Y}$ and $D_{Y\to X}$ determined from repeated experiments. Then, a causal connection was assessed by examining such estimated confidence intervals. The causal link X→Y was detected by mTRGC if the lower one-sided (1−α/2)-confidence interval on $D_{X\to Y}$ did not contain zero. The opposite direction of Y→X was examined analogously, using the lower one-sided (1−α/2)-confidence interval on $D_{Y\to X}$. The results obtained under the (unrealistic) testing condition, from repeated experiments, serve to get an idea of the best possible obtainable results of mTRGC.

The performance of the Granger causality detection methods was evaluated by two rates: false positive (a type I error) and false negative (a type II error). A false-positive rate (FPR) is the proportion of all cases without causal links, where a test result incorrectly indicates the presence of a causal effect. The significance level α is the probability of the type I error. The false-negative rate (FNR) represents the proportion of all existing causal links, where a test result incorrectly failed to detect the causal link. The power of a test is defined as one minus the probability of the type II error. We recall that, in the case of the time-reversed series and unidirectionally connected variables, Y→X was considered the ground true, if it was X→Y for the original, forward series.

## 4. Results

The determined FPRs and FNRs for a model system were averaged according to a condition, sample size, and a testing procedure. The averaged rates of false results are presented in Table 1 for causally independent variables, in Table 2 for unidirectionally connected variables, and in Table 3 for bidirectionally connected variables. It follows from the definition of the mTRGC* that the observed FPR on the time-reversed series is complementary to the observed FPR on the original time series (i.e., their sum equals 100 %) for causally independent variables; the observed FNR on the time-reversed series is complementary to the observed FNR on the original time series for bidirectionally causally connected variables; and the observed FPR, FNR on the original time series are changed vice-versa on the time-reversed series for unidirectionally causally connected variables. Due to the fact that the results obtained by mTRGC* are complementary in this way, the values of the time-reversed series are not shown in the presented tables. The results of GC+mTRGC* obtained on the time-reversed series are not presented in tables either, for more details see Section 4.5.

### 4.1. GC Results

**Figure 1:**
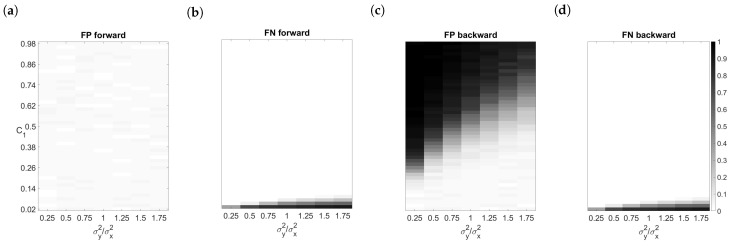
Rates of false detections obtained by GC on time series of length T = 300 generated with normally distributed errors (condition A) for unidirectionally causally connected variables (X→Y): (**a**) false positive rates observed on original time series, (**b**) false negative rates observed on original time series, (**c**) false positive rates observed on time-reversed series, (**d**) false negative rates observed on time-reversed series.

It can be concluded that GC is an exact test for the Granger causality. The presented FPRs obtained on the original time series are very close to the chosen significance level. This is true even for predictive error distributions that are different from the normal distribution which is usually required for the validity of Granger causality analysis. The only exceptions are the false positive results obtained under condition F, see Table 1 and Table 2. Similar FPRs are observed independently of a regression coefficient *a* and of the predictive error variances in the case of causally independent variables. Except for the condition F, the obtained FPRs for unidirectionally connected variables are independent of a value of the connectivity structure control parameter $c_{1}$ and of the predictive error variances, see Figure 1a. The FPRs observed under condition F for unidirectionally connected variables increased as the product of the connection strength $c_{1}$ and the ratio of variance of the driver relative to the recipient increased. If we drew this dependence, we would got a triangle shape similar to the one in Figure 1c which we will speak about later.

The power of GC increases (or equivalently, FNR decreases) with increasing sample size, see Table 2 and Table 3. GC produces false-negative results for small values of the connectivity structure control parameters, $c_{1}$ and $c_{2}$, and is sensitive to heteroscedasticity of predictive error variances. Indeed, FNRs for weakly connected variables are higher if the predictive error variance of the recipient is higher than the predictive error variance of the driver, see Figure 1b.

Let us now look at the results of GC after application to the time-reversed series. It is worth emphasizing that the fitting of a VAR(*p*) on the time-reversed series leads to the problem of endogeneity bias. In that case, the values x(t−p) and y(t−p) are expressed as linear functions of x(t−p+1), x(t−p+2), *…*, x(t), y(t−p+1), y(t−p+2), *…*, y(t) and consequently, the regressors x(t) and y(t) correlate with the predictive error. Then, OLS are biased and that can lead to spurious causal detection as it happened under the condition F for the original time series.

The FPRs obtained under the conditions A–F on the time-reversed series of causally independent variables are similar to those observed on the original time series, see Table 1. Although the presence of instantaneous interactions (condition G) did not pose a complication for correct causal inference on the original time series of causally independent variables, spurious causal identifications occurred after time reversal as consequence of endogeneity, see Table 1.

**Figure 2:**
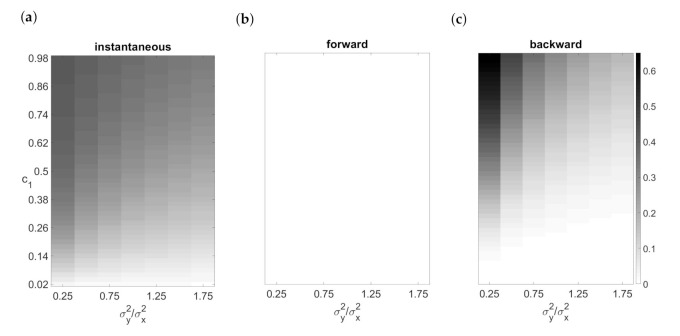
Correlation for time series of length T = 300 generated with independent normally distributed errors (condition A) for unidirectionally connected variables (X→Y): (**a**) correlation of variables, (**b**) correlation of predictive error elements fitted by VAR on original time series, (**c**) correlation (multiplied by −1) of predictive error elements fitted by VAR on time-reversed series.

Similarly, the observed FPRs for the time-reversed series of unidirectionally connected data were larger than the chosen significance level. Elements of the forward predictive errors were uncorrelated on the set of values of connection strength and the ratio of predictive error variances, see Figure 2b. However, a strong correlation structure occurred after time reversal, see Figure 2c. The influences on the dependent variable which were not captured by the model were collected in the predictive error. The endogeneity bias depends on the correlation of the variables (see Figure 2a) and on the ratio of predictive error variances of the variables simultaneously, see Figure 2c.

We can see in Figure 1c that the FPRs for unidirectionally connected variables increase to 1 by increasing the value $c_{1}$ and decreasing the predictive error variance of recipient relative to the driver. The observed FPRs differed between conditions and increased with increasing sample size, due to an inconsistency of endogeneity bias.

On the other hand, non-zero FNRs for the time-reversed series of unidirectionally connected data were observed for small values of $c_{1}$ to a very similar extent as for the original time series, see Table 2, Figure 1b,d.

The endogeneity bias also induced that FNRs obtained on the time-reversed series for bidirectionally connected variables are strictly higher than those observed on the original time series. They are higher for a weak feedback between variables and if the predictive error variance of the recipient is higher than that of the driver.

### 4.2. PEGC Results

PEGC is a conservative test of Granger causality (i.e., the probability of the type I error is smaller than the chosen 0.05/2 significance level), which was found to be sensitive to predictive error distribution and violation of model’s assumptions. Besides, the power of PEGC was much smaller than the power of GC (see Table 1, Table 2 and Table 3).

Similarly to GC, the presence of instantaneous interactions invoked false positive detections after time-reversal. In contrast to GC, FNR obtained on the time-reversed series for unidirectionally connected variables changed under some conditions. The number of detected bidirectional connections on the time-reversed series for unidirectionally connected variables was lower compared to the GC results.

### 4.3. mTRGC Results

The observed FPRs on the original time series equal to 0 % for both causally independent variables and unidirectionally connected variables under all considered situations. It can be concluded that mTRGC is a conservative test for Granger causality and sensitive to predictive error distribution. Similarly, as for GC, false negativity occurs for weakly connected variables. The observed FNRs for unidirectionally connected variables are higher than those obtained by GC, but lower than the sum of FPR+FNR obtained by GC for T=3000 under any conditions. No bidirectional connection was correctly detected by mTRGC (see Table 1, Table 2 and Table 3).

The results obtained on the original time series and on the time-reversed series are very similar, except for the case of causally independent variables and the condition G. If a causal link was detected by mTRGC on the original time series for undirectionally causally connected variables, then opposite causal link was generally detected on the time-reversed series.

### 4.4. mTRGC* Results

The observed FPRs for causally independent variables and the observed FNRs for bidirectionally connected variables are both equal to 50 %, only the FPRs for correlated causally independent variables differ. A causal link was incorrectly detected for causally independent variables. Only the dominant causal link was detected in the case of bidirectionally causally connected variables.

The observed FPRs and FNRs for unidirectionally causally connected variables were similar for a condition and a sample size. Their sum was smaller than the sum of FPR+FNR obtained by GC or mTRGC. Similarly to mTRGC, the larger FPRs and FNRs are observed under condition F.

In the case of unambiguous unidirectionally connected variables, the opposite causal link was detected by mTRGC* after time-reversal. Bidirectional causal connections were incorrectly detected on the time-reversed series for correlated causally independent variables. For the results, see Table 1, Table 2 and Table 3.

### 4.5. GC+mTRGC*

Since the smallest number of false detections (FPR+FNR) for unidrectionally connected variables was obtained by mTRGC*, we proposed to combine mTRGC* with GC. Our intention was to analyze a potential improvement of GC by using the results from the time-reversed series, on the original time series. The observed FPRs were similar to the FPRs obtained by GC for causally independent variables, except under the condition G. Since the highest number of correctly detected absence of a causal link occurred for correlated causally independent variables, the difference was expected. A significant number of the false positive detections by GC was rejected by additional applying mTRGC* for unidirectionally connected variables. Moreover, the observed FNRs by GC for unidirectionally connected variables did not change significantly after applying mTRGC*. As it was expected based on the previous results, many of correctly detected connections by GC for bidirectionally connected variables were rejected after additional application of mTRGC*. For the results, see Table 1, Table 2 and Table 3.

## 5. Discussion

In the case of stochastic data defined by autoregressive models, the change of the direction of causality after the time reversal is investigated by different Granger causality detection methods. The clear effect of a change in the order of cause and effect is widely observed by mTRGC and mTRGC*, while GC and PEGC observe a clear reversal of causality only under specific conditions. Unambiguously opposite direction of causal link was detected by GC and PEGC on the time-reversed series only when the product of the connection strength and the ratio of the predictive errors of the driver relative to the recipient were below a certain level. If it was above that level, bidirectional causal link was mostly detected by GC and PEGC. The bidirectional causal detections after time-reversal of unidirectionally causally connected variables might occur as consequence of the endogeneity bias. Indeed, components of the backward predictive errors were correlated on similar set of values of the connection strength and of the ratio of the predictive error variances for which bidirectional causal connection was detected. The set of values leading to such bidirectional detection even increased with increasing sample size.

Although, in general, the methods based on time-reversal testing suffer from the inability to correctly detect bidirectional connections, they can serve to verify the results of GC. A falsely detected unidirectional causal connection by GC can be rejected by applying mTRGC* additionally. Moreover, the absence of causal link detected by GC on the original series should be detected also on the time-reversed series of uncorrelated causally independent variables.

GC test turned out to be an exact test for Granger causality even for predictive error distributions that are different from the normal distribution. However, the assumption of no-autocorrelated predictive errors was crucial for validity of GC. Our results indicate that, even if a part of the model assumption is violated, under some circumstances, GC can still yield meaningful results. Finally, it should be mentioned that even if the autoregressive model fits the correlation structure in the data, spurious causalities could still arise if some relevant variables are not analyzed. The problem of a hidden confounding variable as well as measurement noise issues were not considered in this work.

## Figures and Tables

**Table 1:** False positive rates (in %) for causally independent (X⊥Y) variables. The results for eight discussed testing procedures (**inv**—results in the time-reversed series) are presented, with the worst (more than 3%) FPR highlighted in bold.

Condition for $\epsilon_{x}$, $\epsilon_{y}$	Sample Size		GC	inv GC	PEGC	inv PEGC	mTRGC	inv mTRGC	mTRGC*	GC +mTRGC*
A	300	FPR	2.6	2.6	0.6	0.6	0	0	**49.9**	2.5
	3000	FPR	2.5	2.5	0.6	0.6	0	0	**49.8**	2.5
B	300	FPR	2.7	2.6	1.1	0.7	0	0	**50.1**	2.6
	3000	FPR	2.5	2.5	1.1	0.6	0	0	**50**	2.5
C	300	FPR	2.6	2.6	0.9	0.6	0	0	**50**	2.5
	3000	FPR	2.5	2.5	0.9	0.6	0	0	**49.9**	2.4
D	300	FPR	2.7	2.6	0.6	0.7	0	0	**50.1**	2.6
	3000	FPR	2.5	2.5	0.6	0.6	0	0	**49.9**	2.4
E	300	FPR	2.7	2.7	2.4	2.3	0	0	**50.1**	2.6
	3000	FPR	2.5	2.5	**3.9**	**3.8**	0	0	**50**	2.5
F	300	FPR	**3.4**	**3.5**	1.7	1	0	0	**50**	**3.4**
	3000	FPR	**3.2**	**3.2**	**5.9**	1.8	0	0	**50**	**3.1**
G	300	FPR	2.6	**17.2**	0.7	**3.2**	0	2.6	**34.7**	2.2
	3000	FPR	2.6	**62.3**	0.6	**27.6**	0	**43.2**	**13.2**	1.1

**Table 2:** False positive rates and false negatives rates (in %) for unidirectionally causally connected (X→Y) variables. The results for eight discussed testing procedures (**inv**—results in the time-reversed series) are presented, with the worst (more than 3%) FPR highlighted in bold.

Condition for $\epsilon_{x}$, $\epsilon_{y}$	Sample Size		GC	inv GC	PEGC	inv PEGC	mTRGC	inv mTRGC	mTRGC*	GC +mTRGC*
A	300	FPR	2.2	**11.7**	0.7	1.8	0	0	**3.4**	0.3
		FNR	10.3	10.3	22.2	22.3	14	14.2	3.5	10.3
	3000	FPR	2.3	**48.1**	0.9	**16**	0	0	0.7	0.1
		FNR	2.5	2.5	6.4	6.5	4	4	0.7	2.5
B	300	FPR	2.2	**12.3**	1.3	2.2	0	0	**3.6**	0.3
		FNR	10.7	10.7	31.6	22	14.8	15.4	3.6	10.7
	3000	FPR	2.3	**47**	1.6	**17.8**	0	0	0.8	0.1
		FNR	2.7	2.7	9.7	6.3	4	4	0.8	2.7
C	300	FPR	2.3	**12.2**	1.1	2	0	0	**3.7**	0.3
		FNR	10.7	10.7	28.2	22.5	15.4	15.4	3.7	10.8
	3000	FPR	2.4	**46.9**	1.3	**17.4**	0	0	0.8	0.1
		FNR	2.6	2.6	8.7	6.5	4	4	0.8	2.7
D	300	FPR	2.3	**8.3**	0.6	1.4	0	0	**4.1**	0.4
		FNR	12	12	26.5	25.4	18	17.2	4.1	12
	3000	FPR	2.4	**40.4**	0.7	**11**	0	0	0.9	0.1
		FNR	3	3	7.9	7.4	4.8	4.8	0.9	3
E	300	FPR	2.4	**6.7**	2.3	**3.8**	0	0	**4.2**	0.4
		FNR	12.5	12.4	21	21.1	17.4	17.4	4.3	12.5
	3000	FPR	2.6	**36.3**	**4**	**19.7**	0	0	1	0.1
		FNR	3.4	3.4	5.9	5.8	5.2	5.2	1	3.4
F	300	FPR	**16.1**	**9.3**	1.9	2	0	0	**5.9**	0.7
		FNR	15.9	15.9	17.7	39.9	24.2	24.4	5.9	16
	3000	FPR	**51.7**	**52.5**	**22.6**	**19.1**	0	0	1.5	0.2
		FNR	4.4	4.4	5.3	12.5	6.2	6.2	1.5	4.5
G	300	FPR	2.4	**34.1**	0.8	**7.6**	0	0	**3.4**	0.3
		FNR	10.7	10	23.6	21.7	15.4	14.2	3.7	10.8
	3000	FPR	2.4	**73.5**	1	**51.2**	0	0	0.7	0.1
		FNR	2.6	2.4	6.7	6.3	4.4	3.8	0.8	2.6

**Table 3:** False negatives rates (in %) for bidirectionally causally connected (X↔Y) variables. The results for eight discussed testing procedures (**inv**—results in the time-reversed series).

Condition for $\epsilon_{x}$, $\epsilon_{y}$	Sample Size		GC	inv GC	PEGC	inv PEGC	mTRGC	inv mTRGC	mTRGC*	GC +mTRGC*
A	300	FNR	9.7	13.7	24.7	28.6	62.4	62	50	50
	3000	FNR	1.8	5.1	5.8	9.1	53	53	50	50
B	300	FNR	12.7	21.5	42.4	34.5	57.9	57.9	50	50.1
	3000	FNR	2	8.2	11	14	52.3	52.6	50	50
C	300	FNR	12.8	21.6	38.3	35.5	57.9	57.5	50	50.1
	3000	FNR	2	8.3	9.3	14.2	51.9	51.9	50	50
D	300	FNR	11.2	15.9	25.5	27.9	58.4	58.4	50	50
	3000	FNR	2.3	6.6	7.2	10.9	52.1	52.1	50	50
E	300	FNR	13	16.4	25.2	28.7	62	62.8	50	50
	3000	FNR	3.3	6.2	5.8	8.7	53	53	50	50
F	300	FNR	33.7	34.4	34.7	61.7	66.2	65.8	50	50.6
	3000	FNR	2.1	9	5.6	21.9	54.5	54.9	50	50
G	300	FNR	10.3	19.9	27.6	34.1	61.3	60.9	50	50
	3000	FNR	2	7.3	6	13.8	53	53	50	50

## Abbreviations

The following abbreviations are used in this manuscript:

AR	Autoregressive model
VAR	Vector autoregressive model
GC	Standard Granger causality test
PEGC	Predictive error test of Granger causality
TRGC	Time-reversed Granger causality test
mTRGC	Modification of time-reversed Granger causality test
mTRGC*	Modification of time-reversed Granger causality (non-statistical) test
FPR	False-positive rate
FNR	False-negative rate
